# Psychophysiological Correlates of the Disposition Effect

**DOI:** 10.1371/journal.pone.0054542

**Published:** 2013-01-23

**Authors:** Marco Goulart, Newton Da Costa, Andre Santos, Emilio Takase, Sergio Da Silva

**Affiliations:** 1 Graduate Program in Economics, Federal University of Santa Catarina, Santa Catarina, Brazil; 2 Graduate Program in Psychology, Federal University of Santa Catarina, Santa Catarina, Brazil; University of Bologna, Italy

## Abstract

We assess the psychophysiological characteristics underlying the disposition effect and find that subjects showing greater disposition effect are those who sweat more and present lower body temperature and heart rate.

## Introduction

In a pioneer field experiment, Lo and Repin [1] studied the decision-making process of professional securities traders by measuring the real-time psychophysiological characteristics–skin conductance response, blood volume pulse, heart rate, electromyographical signals, respiration, and body temperature–during live trading sessions while simultaneously capturing real-time prices from which market events could be detected. To measure the physiological characteristics they used the portable biofeedback equipment ProComp+ data-acquisition unit along with the software Biograph v. 1.2 from Thought Technology. The equipment was attached on each subject’s belt and from which a fiberoptic connection led to a laptop computer equipped with real-time data acquisition software. They found statistically significant differences between skin conductance response and blood volume pulse across periods of high and low volatility; such differences were related to the degree of experience of the traders.

Here we replicate the study of Lo-Repin but this time in the lab using student subjects. The objective is to assess the psychophysiological characteristics underlying the disposition effect to sell winning stocks too early and ride losers too long. We used a more powerful equipment: the electric signal amplifier NeXus-10 from MindMedia along with the software BioExplorer (http://www.mindmedia.nl). We find that subjects showing greater disposition effect are those with higher skin conductance response (those who sweat more), lower body temperature, and lower heart rate.

The disposition effect occurs whenever subjects sell more (less) stocks as the sale price is above (below) either the purchase price or the previous price [2]. Here we consider Odean’s [3] measure of the effect, which mimics real-world market cycles. (Other measures–less appropriate for our study–are the ones of Weber and Camerer [2] and Dhar and Zhu [4]. Odean’s measure considers the actual- and potential trades of investor 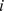 during a sample period. Potential trades refer to stocks in a portfolio that were not sold but that could have been either winners or losers. The proportion of gains realized (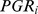) and proportion of losses realized (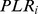) are computed as
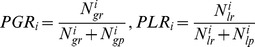
(1)where 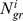 (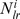) is the number of trades by investor 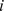 with a realized gain (loss), and 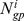 (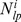) is the number of potential trades for investor 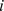 with a gain (loss).

The disposition effect (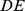) of investor 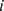 is then
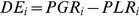
(2)where 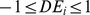. A positive value of 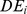 indicates that a smaller proportion of losers is sold compared with the proportion of winners sold, in which case investor 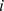 exhibits the disposition effect.

## Methods

The sample was made up of 40 undergraduates in economics, accounting, business administration, and production engineering from the Federal University of Santa Catarina, Brazil. (Lo and Rapin’s experiment involved only ten traders.) All participants signed an informed consent to participate in the research. The research procedures were approved by the Ethics Committee on Human Research of the Federal University of Santa Catarina.

We considered the experiment of Weber and Camerer [2] as a starting point for our experiment design. An investment simulator developed by us called ExpEcon then generated a friendly output in terms of data to be used in calculating the disposition effect. Subjects were approached on an individual basis. Each subject performed three sessions of the experiment (more than in Weber-Camerer’s), but the subjects spent on average less time (30 min) than in Weber-Camerer’s (average time spent = 2 h). As in Weber-Camerer, six stocks were considered and their prices were announced randomly. The only information subjects had were current and past prices of each stock. Some prices had a pre-defined upward or downward trend, a design feature of the experiment conceived by Weber-Camerer intended to remove the possibility of reversal to the mean from the start.

The subjects were incentivized through monetary rewards according to their attendance and performance. Each subject was shown both a handout and a PowerPoint presentation explaining the details of the experiment, including a section relative to the rewards, which reads:

“You are being offered cash prizes totaling R$600.00. The prizes are distributed as follows:

1. Cash prize of R$150.00 for the top performer of the investment simulation (that is, the subject with greater overall return at the end of the simulation), and a prize of R$50.00 for the runner up.
2. Each subject who outperformed the previous one in a row will take part in a raffle of R$200.00.
3. In addition, all participants will take part in a raffle of another R$200.00, regardless of performance.”

The other details of the experiment closely followed Weber-Camerer’s. In particular, the details that matters most in here are exactly those exposed in a previous paper by some of us [5]. The data of time series of the trades can be made available upon request.

## Results and Discussion

The subjects were sorted from bottom to top according to their values for the psychophysiological variables (Table 1). Their correspondent individual disposition effects were then calculated. Table 1 shows the skin conductance response, body temperature and heart rate for the first fourteen subjects with smaller values for these variables (blue font) and for the last fourteen subjects (red font). The mid-value subjects were dismissed.

**Table 1:** Psychophysiological variables and individual disposition effect.

Subject #	Skinconductance response	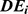	Subject #	Body temperature	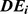	Subject #	Heart rate	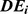
21	−0.1750	−0.0956	30	−0.1094	−0.2574	32	−0.1532	−0.0333
34	−0.0843	0.0000	32	−0.0915	−0.0333	22	−0.0573	0.0251
29	−0.0423	−0.1213	17	−0.0731	0.1463	13	−0.0490	0.0307
17	−0.0200	0.1463	10	−0.0587	−0.3027	15	−0.0453	0.3556
15	−0.0107	0.3556	27	−0.0554	−0.0266	20	−0.0444	−0.0543
4	0.0240	−0.0396	19	−0.0397	−0.2167	3	−0.0400	−0.0804
40	0.0243	−0.1190	15	−0.0385	0.3556	4	−0.0361	−0.0396
6	0.0252	0.1582	2	−0.0341	0.3037	31	−0.0216	0.0451
24	0.0257	0.0250	25	−0.0275	0.1571	18	−0.0091	0.0516
33	0.0324	0.1765	20	−0.0275	−0.0543	1	0.0114	−0.1213
10	0.0347	−0.3027	24	−0.0265	0.0250	6	0.0125	0.1582
20	0.0441	−0.0543	35	−0.0253	−0.1648	21	0.0140	−0.0956
13	0.0517	0.0307	23	−0.0247	−0.1063	36	0.0142	−0.1029
7	0.0745	0.0385	6	−0.0193	0.1582	11	0.0148	0.1134
14	0.2094	0.1595	5	−0.0052	−0.1782	40	0.0899	−0.1190
25	0.2559	0.1571	34	−0.0030	0.0000	30	0.0930	−0.2574
31	0.2864	0.0451	26	−0.0010	−0.0921	35	0.0978	−0.1648
2	0.3089	0.3037	22	0.0001	0.0251	23	0.0979	−0.1063
3	0.3917	−0.0804	14	0.0005	0.1595	10	0.1082	−0.3027
26	0.4026	−0.0921	36	0.0006	−0.1029	16	0.1378	−0.2771
1	0.4718	−0.1213	29	0.0017	−0.1213	27	0.1484	−0.0266
35	0.4754	−0.1648	33	0.0021	0.1765	2	0.1485	0.3037
23	0.4871	−0.1063	11	0.0025	0.1134	19	0.1520	−0.2167
11	0.6834	0.1134	1	0.0030	−0.1213	7	0.1596	0.0385
9	0.8435	−0.0263	13	0.0036	0.0307	14	0.1608	0.1595
32	0.9100	−0.0333	38	0.0052	0.0674	38	0.1683	0.0674
38	0.9636	0.0674	3	0.0055	−0.0804	25	0.1994	0.1571
12	1.5362	0.1824	37	0.0131	−0.2808	29	0.2111	−0.1213

Although there is no significant correlation between the individual variables and the disposition effect in Table 1, there was correlation as we aggregate subjects in two subsamples. The first subsample was made up of the top values of 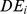, which were summed up to generate an aggregate proportion of gains realized, 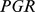. The second subsample was made up of the bottom values of 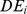, which were summed up to generate an aggregate proportion of losses realized, 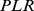. The aggregate disposition effect 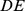 was finally calculated (Tables 2, 3, 4).

**Table 2:** Aggregate disposition effect and skin conductance response.

	Total	Low skin conductance response	High skin conductance response
Number of trades by investor 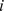 with a realized gain ( 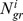 )	202	85	117
Number of trades by investor 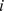 with a realized loss ( 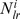 )	73	30	43
Number of potential trades for investor 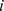 with a gain ( 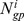 )	1147	567	580
Number of potential trades for investor 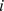 with a loss ( 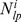 )	578	243	335
Aggregate proportion of gains realized 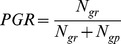	0.1497	0.1304	0.1679
Aggregate proportion of losses realized 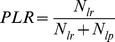	0.1121	0.1099	0.1138
Aggregate disposition effect 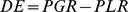	0.03761	0.02048	0.05411
Standard error of the disposition effect	0.01573	0.0231	0.0216
z statistic	2.3912	0.8877	2.5034
p-value	(0.0084)	(0.1874)	(0.0062)

**Table 3:** Aggregate disposition effect and body temperature.

	Total	Low body temperature	High body temperature
Number of trades by investor 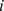 with a realized gain ( 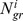 )	209	118	91
Number of trades by investor 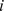 with a realized loss ( 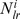 )	94	40	54
Number of potential trades for investor 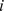 with a gain ( 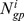 )	1280	607	673
Number of potential trades for investor 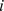 with a loss ( 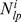 )	578	222	356
Aggregate proportion of gains realized 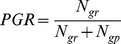	0.1404	0.1628	0.1191
Aggregate proportion of losses realized 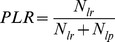	0.1399	0.1527	0.1317
Aggregate disposition effect 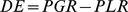	0.00048	0.01009	−0.01260
Standard error of the disposition effect	0.01613	0.0261	0.0204
*z* statistic	0.0299	0.3863	−0.6174

**Table 4:** Aggregate disposition effect and heart rate.

	Total	Low heart rate	High heart rate
Number of trades by investor 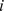 with a realized gain ( 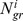 )	191	101	90
Number of trades by investor 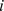 with a realized loss ( 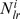 )	90	41	49
Number of potential trades for investor 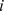 with a gain ( 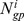 )	1235	567	668
Number of potential trades for investor 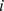 with a loss ( 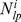 )	582	340	242
Proportion of gains realized 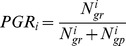	0.1339	0.1512	0.1187
Proportion of losses realized 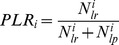	0.1339	0.1076	0.1684
Disposition effect of investor 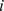 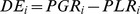	0.0000	0.0436	−0.0497
Standard error of the disposition effect	0.01594	0.0211	0.0249
*z* statistic	0.0008	2.0681	−1.9953
*p*-value	(0.499)	(0.019)	(0.023)

First we compared 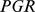 with the variables low skin conductance response and high skin conductance response (Table 2), that is, 85/(85+567) = 0.1304 (low) versus 117/(117+580) = 0.1679 (high) (*z* = −1.929, *p*-value = 0.0537, two-tailed test). Then we did the same for 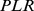, that is, 30/(30+243) = 0.1099 (low) versus 43/(43+335) = 0.1138 (high) (*z* = −0.154, *p*-value = 0.9776, two-tailed test). And finally the 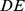 between low and high skin conductance response was calculated, that is, *z* = [*DE*(low) − *DE*(high)]/*Std Error*(*PGR* − *PLR*) = (0.02048−0.05411)/0.01573 = −2.1380 (*p*-value = 0.014, two-tailed test). The disposition effect appeared in the entire sample (*DE* >0) (*p*-value = 0.008) and also in the subsample with high skin conductance response (*p*-value = 0.006). The variable *DE* was positive largely because *PGR* of the subjects with high skin conductance response was high, since that *PLR* remained unchanged.

Then the analysis was repeated to consider the variable body temperature (Table 3). First we compared 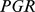 with the variables low body temperature and high body temperature, that is, 118/(118+607) = 0.1628 (low) versus 91/(91+673) = 0.1191 (high) (*z* = 2.42, *p*-value = 0.0154, two-tailed test). Then we did the same for 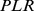, that is, 40/(40+222) = 0.1527 (low) versus 54/(54+356) = 0.1317 (high) (*z* = 0.764, *p*-value = 0.4449, two-tailed test). And finally the 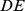 between low and high body temperature was calculated, that is, *z* = [*DE*(low) − *DE*(high)]/*Std Error*(*PGR* − *PLR*) = (0.01009+0.01260)/0.01613 = 1.4067 (*p*-value = 0.0798, two-tailed test). The disposition effect was found to be greater the lower the body temperature, at the 10 per cent significant level (however, *DE* was not significant at 5 per cent). This was caused by a high value for *PGR*.

As for the variable heart rate (the number of heartbeats per minute) the disposition effect did not occur for the entire sample (*DE* <0, *p*-value = 0.499) probably because of the importance of the subsample of subjects with higher heart rate (*DE* <0, *p*-value = 0.023). However, the disposition effect appeared in the subjects with lower heart rate (*DE* >0, *p*-value = 0.019). The *PGR* of this group surpassed that of the higher heart rate group and also the *PLR* of the lower heart group felt below that of the higher heart rate group (Table 4).

What do these results mean? Why should we care? The result that a greater disposition effect is observed for those subjects who sweat more can be compared with the classical study of Bechara, Damasio, Tranel and Damasio [6]. Such study shows that anticipation of riskier outcomes leads to more skin conductance response. The most common explanation for the disposition effect is based on Kahneman and Tversky’s prospect theory, namely that people are risk-averse over gains and risk-seeking over losses. Possibly this explanation makes sense in light of our results (those who sweat more are more likely to keep losing assets) combined with Bechara, Damasio, Tranel and Damasio’s (those who sweat more are anticipating a riskier outcome).

However, some may claim that our result can perhaps still be consistent with rational mean-reversion as an explanation for the disposition effect. For example, Brooks, Capra and Berns [7] find that for decisions below the purchase price, a greater disposition effect is correlated with a blunted ventral striatum response to upticks in value in some individuals. And activity in such brain region scales with both the expected and subjective value of stimuli. The ventral striatum plays a role in signaling reward-prediction errors and thus this blunted response is consistent with meeting an expectation of an uptick towards the mean. We add that if such stimuli are physiologically accompanied with sweat, then our result that those who sweat more show greater disposition effect can also be compatible with the mean-reversion hypothesis.

Body temperature regulation involves the integration of autonomic, motor, and endocrine responses. The temperatures of different parts of the body are related to certain cognitive and emotional contents of a task or stimuli. For example, hand skin temperature decreases with threatening and unpleasant tasks [8]. If the threatening task produces the anticipation of a riskier outcome, then the finding in [8] is complementary to Bechara, Damasio, Tranel and Damasio’s discussed above. Sweating more and having lower body temperature are thus compatible is such a situation. This is thus in line with our result that those who presented lower body temperature were more likely to keep losing assets.

What about our finding that a lower heart rate is associated with those subjects showing the disposition effect? Does this make sense? We think so. Human decision-making is under the constant influence of two separate systems, one automatic and fast and another effortful and slow. And the activities of the effortful system are associated with dilated pupils and an accelerated heart rate [9]. As it happens, visual and cognitive illusions–such as the disposition effect–occur in the realm of the automatic system. Thus, it comes as no surprise that subjects showing the disposition effect are also those with relatively lower heart rate.

### Conclusions

We assessed the psychophysiological characteristics underlying the disposition effect for 40 Brazilian undergraduates. From the variables of the pioneer study by Lo and Repin [1], namely skin conductance response, blood volume pulse, heart rate, electromyographical signals, respiration, and body temperature, we found three of them related to the disposition effect, which was tracked using the Weber and Camerer [2] experiment as a benchmark along with an investment simulator developed by us. Thus, our analysis revealed that the subjects showing greater disposition effect were those who sweat more and presented lower body temperature and heart rate.

## References

[1] Lo AW, Repin DV (2002) The psychophysiology of real-time financial risk processing. Journal of Cognitive Neuroscience 14: 323–339. 11970795 10.1162/089892902317361877

[2] Weber M, Camerer CF (1998) The disposition effect in securities trading: An experimental analysis. Journal of Economic Behavior & Organization 33: 167–184.

[3] Odean T (1998) Are investors reluctant to realize their losses? Journal of Finance 53: 1775–1798.

[4] Dhar R, Zhu N (2006) Up close and personal: Investor sophistication and the disposition effect. Management Science 52: 726–740.

[5] Da Costa Jr N, Mineto C, Da Silva S (2008) Disposition effect and gender. Applied Economics Letters 15: 411–416.

[6] Bechara A, Damasio H, Tranel D, Damasio AR (1997) Deciding advantageously before knowing the advantageous strategy. Science 275: 1293–1294. 9036851 10.1126/science.275.5304.1293

[7] Brooks AM, Capra CM, Berns GS (2012) Neural insensitivity to upticks in value is associated with the disposition effect. NeuroImage 59: 4086–4093. 22079448 10.1016/j.neuroimage.2011.10.081 PMC3288460

[8] Rimm-Kaufman SE, Kagan J (1996) The psychological significance of changes in skin temperature. Motivation and Emotion 20: 63–78.

[9] Kahneman D (2011) Thinking, fast and slow. New York: Farrar, Straus and Giroux. 512 p.

